# Artificial Neural Network and Response Surface Methodology Based Analysis on Solid Particle Erosion Behavior of Polymer Matrix Composites

**DOI:** 10.3390/ma13061381

**Published:** 2020-03-18

**Authors:** Sundeep Kumar Antil, Parvesh Antil, Sarbjit Singh, Anil Kumar, Catalin Iulian Pruncu

**Affiliations:** 1 Department of Soil and Water Engineering, College of Agricultural Engineering and Technology, CCS HAU Hisar, Haryana 125004, India; sundeepkumar@hau.ac.in; 2 Department of Basic Engineering, College of Agricultural Engineering and Technology, CCS HAU Hisar, Haryana 125004, India; parveshantil@hau.ac.in; 3 Department of Mechanical Engineering, Punjab Engineering College, Chandigarh 160012, India; sarb1234.iitroorkee@gmail.com; 4 Department of Farm Machinery and Power Engineering, College of Agricultural Engineering and Technology, CCS HAU Hisar, Haryana 125004, India; anil_saroha@rediffmail.com; 5 Department of Mechanical Engineering, School of Engineering, University of Birmingham, Birmingham B15 2TT, UK; 6 Department of Mechanical Engineering, Imperial College London, Exhibition Rd., London SW7 2AZ, UK

**Keywords:** artificial neural network, erosion, glass fibers, polymer matrix composites, response surface methodology

## Abstract

Polymer-based fibrous composites are gaining popularity in marine and sports industries because of their prominent features like easy to process, better strength to weight ratio, durability and cost-effectiveness. Still, erosive behavior of composites under cyclic abrasive impact is a significant concern for the research fraternity. In this paper, the S type woven glass fibers reinforced polymer matrix composites (PMC_s_) are used to analyze the bonding behavior of reinforcement and matrix against the natural abrasive slurry. The response surface methodology is adopted to analyze the effect of various erosion parameters on the erosion resistance. The slurry pressure, impingement angle and nozzle diameter, were used as erosion parameters whereas erosion loss, i.e., weight loss during an erosion phenomenon was considered as a response parameter. The artificial neural network model was used to validate the attained outcomes for an optimum solution. The comparative analysis of response surface methodology (RSM) and artificial neural network (ANN) models shows good agreement with the erosion behavior of glass fiber reinforced polymer matrix composites.

## 1. Introduction

The demand for polymer matrix composites (PMCs) has been increased significantly in recent years for industrial and household’s applications [1,2,3,4]. The lightweight, better strength and economically affordability made them highly recommended material for the shed in coastal as well as in desert areas. However, the constant abrasive attack in the form of storm and slurry mixed winds causes surface degradation [5,6]. The continuous bombardments of these particles weaken the bonding strength, which causes material failure. This constant bombardment degrades the surface and reduces the material’s life [7]. The erosion on the surface initiates with imprints of minute cracks induced due to stress caused by continuous slurry impact. The components made up of PMCs in aircraft’s outer structure faces surface erosion because of the existence of highly abrasive particles in the air [8,9]. The erosion of fibrous composites depends upon various process parameters such as impacting particles, angle of impingement, velocity, etc. Tewari et al. [10] reported that erosion of fibrous composites is significantly influenced by fiber orientation because unidirectional fiber orientation exhibits semi ductile erosion behavior. Pool et al. [11] reported that polymer composite shows brittle erosion behavior whereas permanent aramid fiber epoxy shows quasi ductile behavior. The increase in weight fraction of glass fiber as reinforcement can convert ductile erosion behavior into brittle behavior [12]. The effect of particle size during erosion is also analyzed and reported that erosion rate of the composite surface is directly proportional to the size of impacting particle and sometimes becomes zero by using particle size of the range of 2 μm [13]. Miyazaki et al. [14] revealed that fiber-reinforced polymers (FRPs) having treated fibers exhibits better resistance against erosion than non-treated fibers because of higher interfacial strength between matrix and reinforcement. The angle of impingement significantly affects the erosion rate of the composite surface. Rajesh et al. [15] revealed that the impact of an abrasive particle at an oblique angle (30°) is much more influencing than the normal impact angle (90°). The optimization of process parameters for tribological behavior has been performed by various scientists using several techniques like the Taguchi’s methodology [16], response surface methodology [17], artificial neural network [18], genetic algorithm [19], etc. Response surface methodology (RSM) is employed as an optimization tool for the modeling and optimization of single as well as multi-objective optimization [20]. In recent times, several research investigations have been performed based on RSM, which consist of empirical finite element models [21]. Generally, a high range of experimental runs is required for the higher-order RSM models. However, this limitation can be avoided by introducing an artificial neural network (ANN). An artificial neural network (ANN) works on the computational method to mimic the neurological indulgence ability of the human intellect [22]. Human awareness has been simulated by ANN in an assessment and depicted implications when offered with loud, complex, unnecessary and constrained confirmation. Before implementing ANN, partial preceding expectations are mandatory about the process beneath the analysis. The ANN can assess any practical function arbitrarily. The capability of ANN to investigate and rationalize the performance of any complicated and non-linear process makes ANN important modeling tool [23,24]. Several researchers have implemented the collective analysis on RSM and ANN. Josh et al. [25] equated RSM and ANN models for mining of artemisinin. Patel et al. [26] forecast surface roughness during roller burnishing by using RSM and ANN models. Lipinski et al. [27] used artificial neural networks to model surface roughness and grinding forces during the grinding process. Song et al. [28] combined ANN with RSM to investigate cutting forces during laser-assisted machining of fused silica.

The erosion of composite surface comes out to be a significant concern for the components, which are continuously exposed to abrasive and dusty environment. The erosion of composite surface depends upon several factors, which include the velocity of erodent, impact distance, erodent size, type nature, etc. To analyze the effect of various parameters, which influences the erosion of composites, the present paper deals with the erosion of polymer composites using ANN and RSM. The experiments were planned as per response surface methodology by considering slurry pressure, nozzle diameter and impingement angle as input erosion parameters as shown in Table 1.

## 2. Materials and Methods

**Figure 1:**
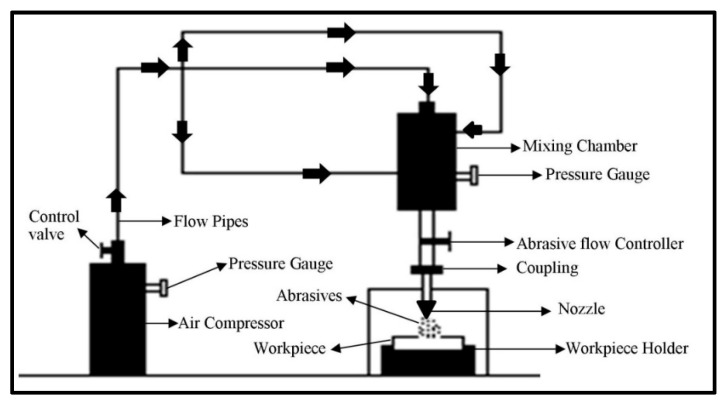
Schematic of the developed solid erosion test setup.

**Figure 2:**
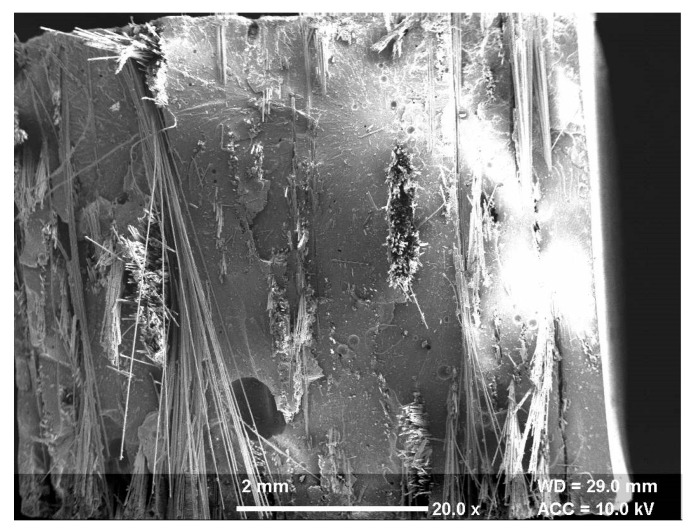
SEM of a cut sectional view of a polymer matric composite (PMC).

**Figure 3:**
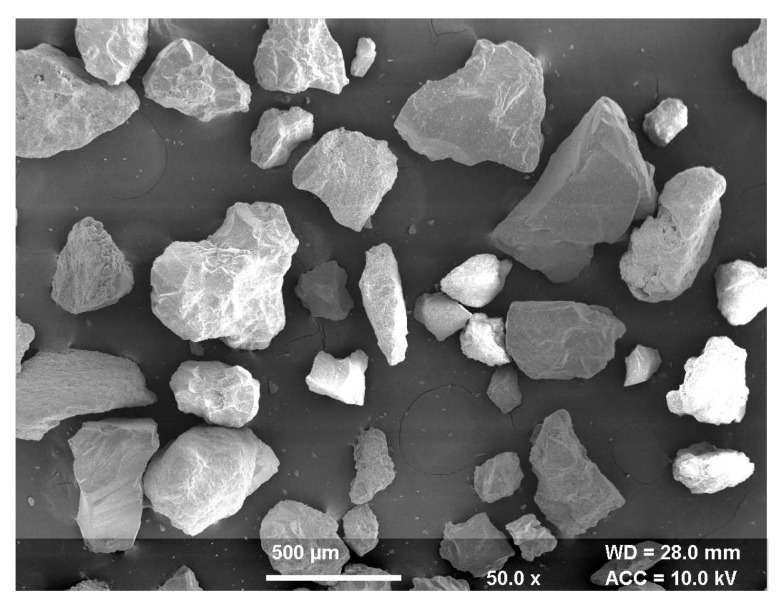
SEM of river sand particles.

Erosion of the composite surface is a complex material phenomenon in which several controlled/uncontrolled parameters collectively affect output quality characteristics. In this paper, statistical analysis of different process parameters for wear behavior of the hybrid polymer matrix composite was studied using the response surface methodology [29]. The box Behnken design based experimental plan was used to study the optimal parametric combination. The experimentation was performed on the developed solid particle erosion test setup (Figure 1) based on the ASTM G76 standard. The polymer matrix composite reinforced with S glass fibers was used as a workpiece material. The river sand particles were mixed with air to bombard on the workpiece as abrasive slurry. The SEM of the cut sectional view of the fabricated composite is shown in Figure 2. The figure clearly shows the different layers of fibers bonded with the polymer matrix. The morphology of the abrasive river sand particles is shown in Figure 3. The magnified images of the sand particles depict that particles possess tapered and sharp edges, which will affect the erosion phenomenon. To keep an eye on erosion behavior, the electronics weighing machine with a least count of 0.0001 g was used to record weight loss. To make the data analysis simple, the ranges were coded based on experimental runs. The point of optimality was chosen at 0 levels. The coded values were determined as follows [30]:

(1) $$X_{k}=\frac{N_{k}\;-\;N_{0}}{N_{1}\;-\;N_{0}}$$

Here N_1_, and N_o_ are values at level 1, and level 0 whereas N_K_ is actual parametric value to level interest. 

The observed experimental results are shown in Table 2. A developed mathematical model based on RSM for correlation of erosion in terms of coded parameters is as follows:

Erosion = 2.3128 + 0.00825 × A − 0.0045 × B + 0.062 × C + 0.00625 × AB + 0.00025 × AC − 0.00225 × BC + 0.010475 × A2 + 0.013975 × B2 − 0.276025 × C2(2)

## 3. Response Surface Methodology

Response surface methodology (RSM) explores the association among numerous process parameters with the response parameter. The observational model is generated by using f numerical and geometric techniques. The generated model is used to improve the response reaction, which is prejudiced by several input parameters. In this paper, the Box–Behnken design was adopted for experimental planning. During RSM, a quantifiable system of association among input parameters and response parameter could be stated as

Y = ϕ(P, N, I)(3)

Here Y is anticipated response and ɸ is response function. For the analysis, a second-order polynomial regression model, which is called a quadratic model, can be written as

(4) $$Y=b_{0}\;+\;\overset{k}{\underset{i=1}{\sum }}b_{i}x+\overset{k}{\underset{i=1}{\sum }}b_{i}x^{2}+\underset{i<j}{\sum }b_{\mathrm{ij}}x_{i}x_{j}$$

The term b_0_ and b_i_ are second-order regression coefficients and b_ii_ and b_ij_ represents a quadratic effect. K represents several machining parameters and x_i_ and x_j_ represents terms, which deal with the effect of machining parameters.

## 4. Artificial Neural Network

**Figure 4:**
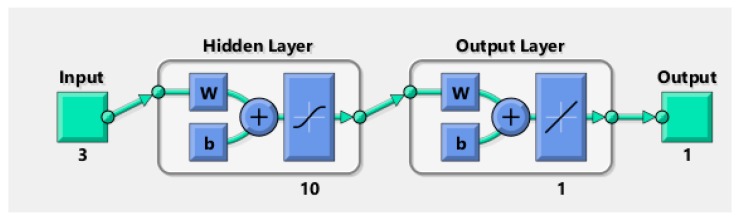
Neural network for input and output.

Artificial neural network (ANN) model is an algebraic model that spontaneously approximates the ability of conventional neural systems. A multilayer perceptron (MLP) was generated in through three input neurons viz. slurry pressure, nozzle diameter and impingement angle, neurons as hidden layers, and target neuron representing the erosion loss. The eccentricity of forecasts from investigational results was reduced by neurons essential in the hidden layer and investigated by a trial. A minimum of ten neurons was obligatory to construct the most recent model using the data accessible, and the development of new neurons presented the probability of over-fitting the model. A cumulative of 90% of investigative consequences was used to formulate the model, with the remaining outcomes, divided justifiably between model consent and testing. The procedure of deciphering fitting problems requires a neural network to plot between input statistics and a set of numeric targets (Figure 4).

During the training phase, the process starts by providing input (data) into the input nodes of the neural network. Then, the feed will be forwarded to the present output on the output nodes of the network. If a similar input is feed in the network, the small error will be generated. In every attempt of training data, the overall error of the network can be measured. The training phase will complete after attaining the best possible solution. The accuracy of the prediction can be influenced by the ANN parameter, i.e., the number of hidden layers. The single hidden layer can estimate the function, which comprises of continuous mapping from one finite space to the adjacent whereas multiple hidden layers signify the arbitrary decision boundary to arbitrary accuracy with a rational activation function. Additionally, multiple hidden layers can estimate any suave mapping to any precision. If there is no hidden layer present, the network will show a discrete linear function.

## 5. Results

### 5.1. Parametric Evaluation through RSM

**Figure 5:**
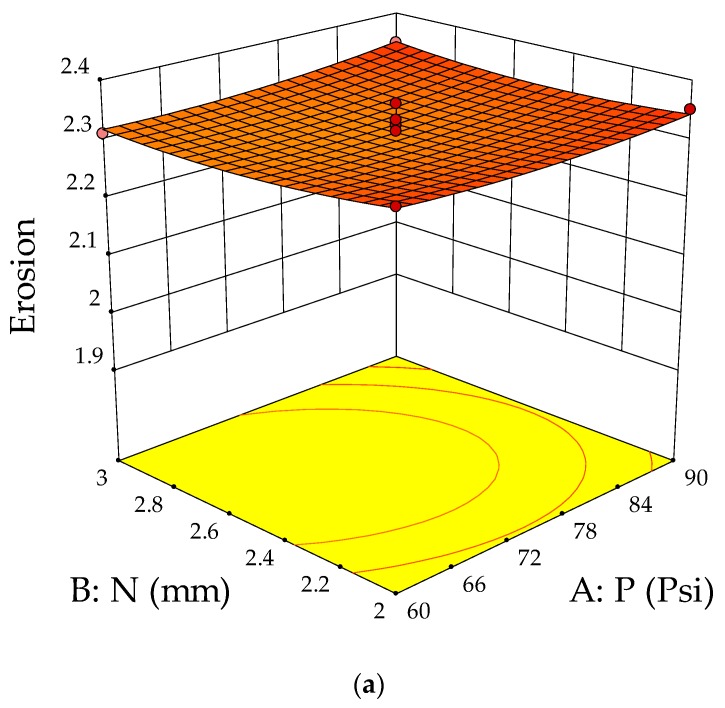

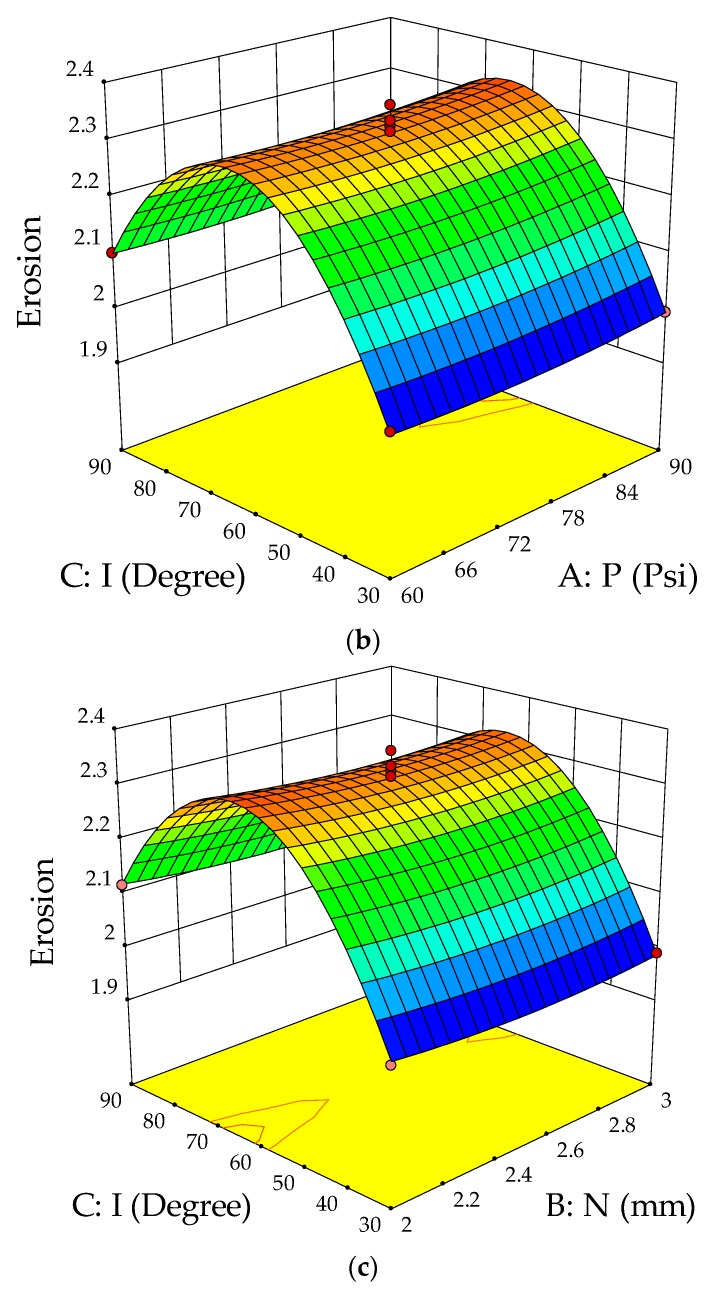
(**a**) Response surface plots for slurry pressure and nozzle diameter on erosion. (**b**) Response surface plots for slurry pressure and impingement angle on erosion. (**c**) Response surface plots for nozzle diameter and impingement angle on erosion.

The ANOVA test was conducted to validate the suitability of developed models for creating a link between the erosion parameters and response. The analysis of variance for erosion is depicted in Table 3. From the ANOVA table, it is clear that the impingement angle was the most significant erosion parameter for the solid particle jet erosion process. The combined effect of machining parameters on erosion is shown in Figure 5a–c. Figure 5a shows the influence of slurry pressure and nozzle diameter on the erosion rate at a constant impingement angle of 60°. The interaction of parameters predicts that at a constant impingement angle of 60°, the erosion was highest with a slurry pressure of 75 Psi and a nozzle diameter of 2.5 mm. The combined effect of the nozzle diameter and impingement angle at constant slurry pressure of 75 Psi is shown in Figure 5b. The results predict that erosion increased with an increase in the impingement angle from 30 to 60° but started decreasing in the next level. During this phase, the nozzle diameter had the least effect on erosion loss. Figure 5c shows the interaction effect of slurry pressure and impingement angle on erosion at a constant nozzle diameter of 2.5 mm. The surface plot indicates that a medium level of impingement angle with maximum slurry pressure produced higher erosion over the composite surface.

### 5.2. Modeling Through ANN

**Figure 6:**
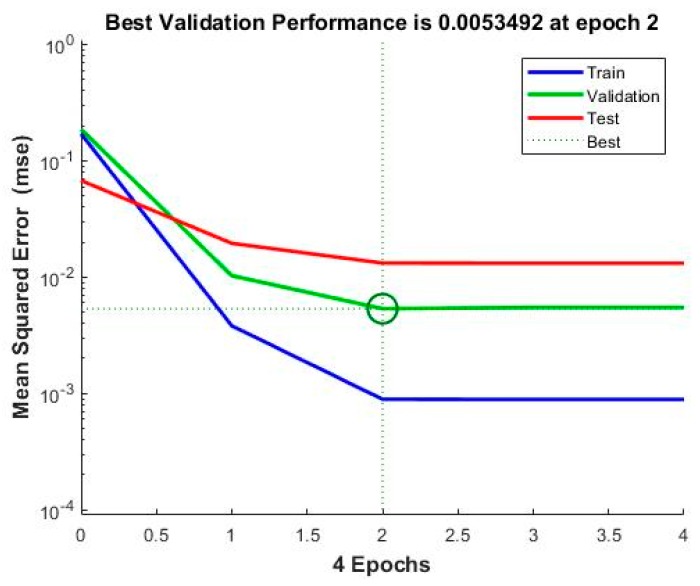
Performance curve.

**Figure 7:**
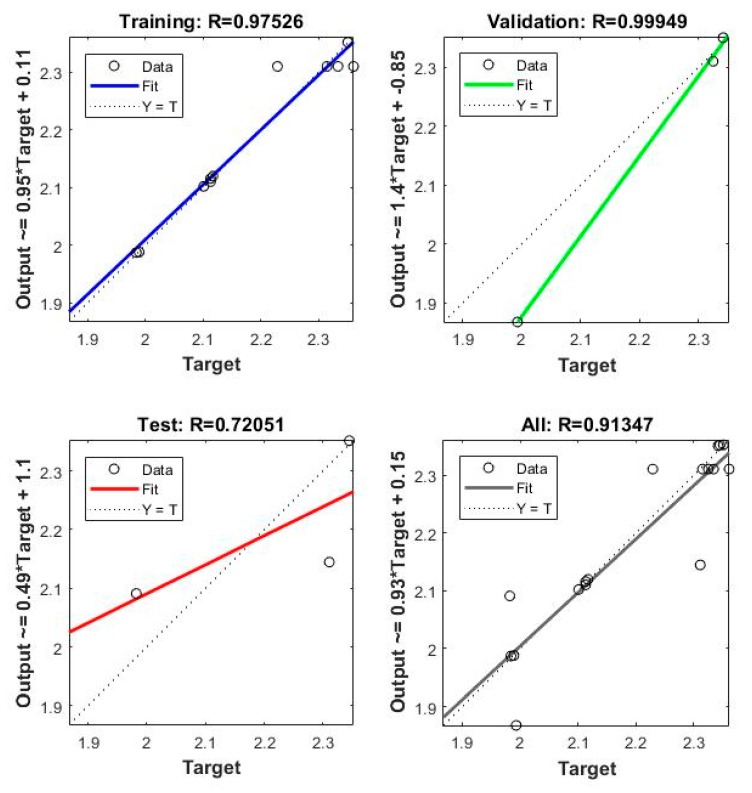
Regression graphs for erosion.

**Figure 8:**
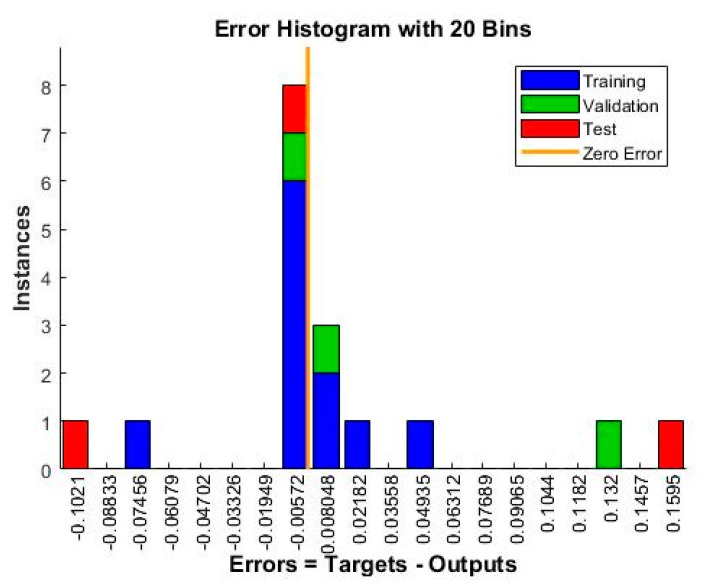
Error histogram chart.

The model is incited by the observance that can acquire within the prospect of a trainer. During modeling, the trainer specifies the precise responses to the contributing parameters. The neural model can equally gain without a trainer, reliant on the criteria of self-association. The neural model illustration is principally established on technical models. The model can be reflected as a model of neurons arranged in limited layers to be explicit the contributing parameters, hidden neurons and response. This methodology has arisen as an innovative and extensive model, which can be regulated to assess any mapping with enough perceptiveness of layers and number of neurons. Table 4 shows the result obtained from the planning of the model. Mean squared error (MSE) characterizes the mediocre squared divergence amid response and targets. The lesser approximations of MSE are healthier, and zero shows no error. Regression (R) values express the linking amid response and goals. An R-value of 1 indicates a sensible bond, and 0 indicates an uneven association. Although, the approximations of MSE and R are nearby zero and one individually. This suggests the curve fitting was exact in the control. The predictable network model was planned equitably, and its presentation was scheduled to validate if any alteration to be prepared for the training practice. Figure 6 shows the performance curve for the developed models. The models show the best validation point occurred at the second iteration. Figure 7 determines the generated regression plots for the testing, training and validation procedure. The obtained result shows the linkage between target and response from the model. Figure 8 exhibits the error histogram for the organized neural model.

## 6. Discussion

**Figure 9:**
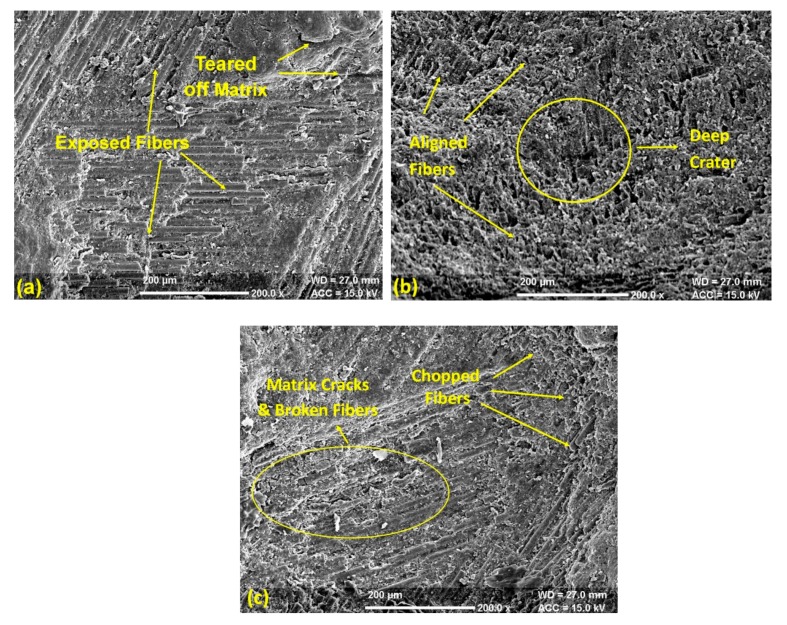
(**a**) SEM of erosion at a 30° impingement angle, (**b**) SEM of erosion at a 60° impingement angle and (**c**) SEM of erosion at a 30° impingement angle.

The overall analysis shows that the impingement angle of striking the slurry influenced the erosion process more significantly than slurry pressure and nozzle diameter. The available trends show that the present composite material could be classified in the semi ductile category because these materials show the highest erosion in the range of 45–60° for the impingement angle [31]. Additionally, the essential factor in controlling the erosion behavior is the nature of the erodent particle. The irregular shape and size of erodent particles induce high erosion in polymer matrix composites [32]. The form of striking erosive particles profoundly influences the nature of deformation of the surface. The round edge particles induce plastic deformation, whereas sharp and hard particles exhibit brittle deformation of the surface [33]. The state of the composite surface at a variable impingement angle is shown in Figure 9a–c. At the impingement angle of 30°, the abrasive slurry chipped off and tore down the composite surface, which made glass fibers visible as shown in Figure 9a. The slurry impact somehow degraded the upper layer, but the strong interfacial bond strength kept the fibers and matrix closely bonded. However, with the change in the impingement angle to 60°, the degradation rate of the primary layer of matrix increased and brought reinforcement into direct contact of the abrasive slurry. The exposed reinforcement aligned in one direction and resulted in high erosion loss. The upper surface damage at the impingement angle of 60° is visible in Figure 9b. The further increase in the impingement angle to 90° decreases the horizontal component [34]. This decrease in the horizontal component decreased the cutting rate of fibers and matrix as compared to erosion at 60º because the increased vertical composite produced a harrier surface, which significantly reduced the erosion rate (Figure 9c). Although few broken fibers and matrix cracks were visible over the surface, still the matrix was uniformly bonded with fiber reinforcement.

**Figure 10:**
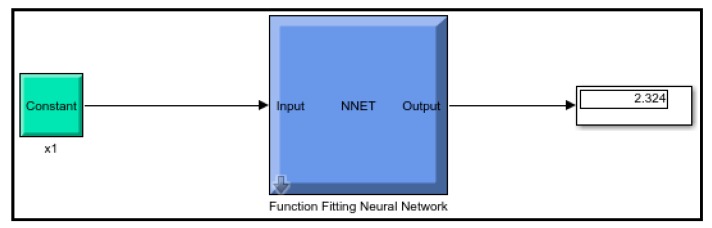
Developed ANN model.

The validation and test outcomes additionally demonstrated the R-value that was more prominent than 0.90. The comparative analysis of the test and the anticipated value of erosion are shown in Table 5. The obtained results for erosion shows 0.043% deviation concerning results obtained from RSM (Figure 10). The attained conclusions designated a prodigious pact between neural system anticipation and test validation standards.

## 7. Conclusions

The following conclusions were drawn from the present study:

1. The erosion during the solid particle impact is deeply affected by the impingement angle. The maximum erosion occurred at an angle of 60°, which means the composite lay in the category of semi ductile materials.
2. From the ANOVA table for erosion, the most significant and influential parameter was found to be the impingement angle. Additionally, the generated quadratic models were suitably fitted with investigational results.
3. The SEM analysis of the river sand particles shows the irregular and sharp conical edges, which were responsible for the high erosion rate.
4. The SEM analysis of composite surface shows that the impingement angle of 60° degraded the upper layer of the composite very finely and exposed the fibers, which caused an excess material loss in comparison to a 30° and 90° impingement angle.
5. MATLAB’s neural network fitting app was used for generating a network model, which produced good comparative results by using hidden layers and neurons. The developed model showed 0.43% deviation with the results obtained from RSM based model.
6. The multiple hidden layers signified an arbitrary decision boundary to arbitrary accuracy with rational activation function and provided precise result with minimal deviation in comparison to the RSM model.
7. The comparative analysis showed that the ANN model could be used proficiently for the validation of single response optimized results obtained during solid particle erosion of polymer matrix composites.

## Figures and Tables

**Table 1:** Machining parameters and levels.

Symbol	Erosion Parameters	Level 1	Level 2	Level 3
P	Slurry Pressure (Psi)	60	75	90
N	Nozzle Diameter (mm)	2.0	2.5	3.0
I	Impingement Angle (°)	30	60	90

**Table 2:** Controllable parameters and results.

Exp. No.	Slurry Pressure (Psi)	Coded Value	Nozzle Diameter (mm)	Coded Value	Impingement Angle (⸰)	Coded Value	Mean Erosion (mg/min)
1	75	0	2.5	0	60	0	2.229
2	75	0	2.5	0	60	0	2.325
3	75	0	2	−1	90	1	2.117
4	75	0	3	1	90	1	2.113
5	90	1	2	−1	60	0	2.351
6	60	−1	3	1	60	0	2.311
7	75	0	2	−1	30	−1	1.984
8	75	0	2.5	0	60	0	2.315
9	90	1	2.5	0	30	−1	1.993
10	60	−1	2.5	0	90	1	2.101
11	90	1	3	1	60	0	2.345
12	75	0	2.5	0	60	0	2.334
13	60	−1	2	−1	60	0	2.342
14	90	1	2.5	0	90	1	2.113
15	60	−1	2.5	0	30	−1	1.982
16	75	0	3	1	30	−1	1.989
17	75	0	2.5	0	60	0	2.361

**Table 3:** ANOVA for the erosion analysis.

Source	Sum of Squares	df	Mean Square	F Value	*p*-Value	Remarks
Model	0.35	9	0.039	26.83	0.0001	Significant
A-P	5.445 × 10^−4^	1	5.445 × 10^−4^	0.37	0.5607	
B-N	1.620 × 10^−4^	1	1.620 × 10^−4^	0.11	0.7488	
C-I	0.031	1	0.031	21.06	0.0025	
AB	1.563 × 10^−4^	1	1.563 × 10^−4^	0.11	0.7531	
AC	2.500 × 10^−7^	1	2.500 × 10^−7^	0.001712	0.9899	
BC	2.025 × 10^−5^	1	2.025 × 10^−5^	0.014	0.9096	
A2	4.620 × 10^−4^	1	4.620 × 10^−4^	0.32	0.5913	
B2	8.223 × 10^−4^	1	8.223 × 10^−4^	0.56	0.4774	
C2	0.32	1	0.32	219.73	<0.0001	
Residual	0.010	7	1.460 × 10^−3^			
Lack of Fit	2.710 × 10^−4^	3	9.033 × 10^−5^	0.036	0.9894	Not Significant
Pure Error	9.949 × 10^−3^	4	2.487 × 10^−3^			
Cor Total	0.36	16				

**Table 4:** Mean squared error and R values from model.

Phases	Sample	MSE	R
Training	11	3.18611 × 10^−4^	9.93792 × 10^−1^
Validation	3	5.16810 × 10^−3^	9.61107 × 10^−1^
Testing	3	8.59712 × 10^−3^	7.60270 × 10^−1^

**Table 5:** Comparative analysis on response surface methodology (RSM) and artificial neural network (ANN).

Model	Parametric Values	Erosion	Deviation
RSM	[75;2.5;60]	2.325	0.43%
ANN	[75;2.5;60]	2.324	

## References

[1] Sathish kumar T.P., Naveen J., Satheesh S. (2014). Hybrid fiber reinforced polymer composites—A review. J. Reinf. Plast. Compos..

[2] Singh M., Singh S. (2019). Electrochemical discharge machining: A review on preceding and perspective research. J. Eng. Manuf..

[3] Rajak D.K., Pagar D.D., Kumar R., Pruncu C.I. (2019). Recent progress of reinforcement materials: A comprehensive overview of composite materials. J. Mater. Res. Technol..

[4] Antil P., Singh S., Prakash C., Singh S., Pruncu C. (2019). Metaheuristic approach in machinability evaluation of SiCp/Glass fiber reinforced PMCs during ECDM. Meas. Control.

[5] Antil P., Singh S., Manna A. (2018). SiCp/Glass fibers reinforced epoxy composites: Wear and erosion behaviour. Indian J. Eng. Mater. Sci..

[6] Antil P., Singh S., Manna A. (2018). Genetic algorithm based optimization of ECDM process for polymer matrix composite. Mater. Sci. Forum.

[7] Friedrich K., Pei X.Q., Almajid A. (2013). Specific erosive wear rate of neat polymer films and various polymer composites. J. Reinf. Plast. Compos..

[8] Qian D.N., Bao L.M., Takatera M., Kemmochi K., Yamanaka A. (2010). Fiber-reinforced polymer composite materials with high specific strength and excellent solid particle erosion resistance. Wear.

[9] Patnaik A., Satapathy A., Chand N., Barkoula N.M., Biswas S. (2010). Solid particle erosion wear characteristics of fiber and particulate filled polymer composites: A review. Wear.

[10] Tewari U.S., Harsha A.P., Hager A.M., Friedrich K. (2003). Solid particle erosion of carbon fibre and glass fibre-epoxy composites. Compos. Sci. Technol..

[11] Pool K.V., Dharan C.K.H., Finnie I. (1986). Erosive wear of composite-materials. Wear.

[12] Tsuda K., Kubouchi M., Sakai T., Saputra A.H., Mitomo N. (2006). General method for predicting the sand erosion rate of GFRP. Wear.

[13] Dundar M., Intal O.T., Stringer J. (1999). The effect of particle size on the erosion of a ductile material at the low particle size limit. Wear.

[14] Miyazaki N., Hamao T. (1996). Effect of interfacial strength on erosion behavior of FRPs. J. Compos. Mater..

[15] Rajesh J.J., Bijwe J., Venkataraman B., Tewari U.S. (2004). Effect of impinging velocity on the erosive wear behaviour of polyamides. Tribol. Int..

[16] Antil P. (2018). Experimental analysis on wear behavior of PMCs reinforced with electroless coated silicon carbide particulates. Silicon.

[17] Padmaraj N.H., Vijaya K.M., Dayananda P. (2019). Experimental investigation on solid particle erosion behaviour of glass/epoxy Quasi-isotropic laminates. Mater. Res. Express.

[18] Subhrajit R.A.Y., Rout A.K., Sahoo A.K. (2018). A study on erosion performance analysis of glass-epoxy composites filled with marble waste using artificial neural network. UPB Sci. Bull. Ser. B.

[19] Patnaik A., Satapathy A., Mahapatra S.S., Dash R.R. (2008). A modeling approach for prediction of erosion behavior of glass fiber–polyester composites. J. Polym. Res..

[20] Montgomery D.C. (1997). Design and Analysis of Experiments.

[21] Feng L., Wei X., Zhao Y.Z., Jing Z., Zheng T. (2012). Analytical prediction and experimental verification of surface roughness during the burnishing process. Int. J. Mach. Tools Manuf..

[22] Sagbas A. (2011). Analysis and optimization of surface roughness in the ball burnishing process using response surface methodology and desirabilty function. Adv. Eng. Softw..

[23] Zain A.M., Habibollah H., Safian S. (2010). Prediction of surface roughness in the end milling machining using artificial neural network. Expert Syst. Appl..

[24] Erzurumlu T., Hasan O. (2007). Comparison of response surface model with neural network in determining the surface quality of moulded parts. Mater. Des..

[25] Pilkingtona J.L., Prestonb C., Gomesa R.L. (2014). Comparison of response surface methodology (RSM) and artificialneural networks (ANN) towards efficient extraction of artemisininfrom Artemisia annua. Ind. Crop. Prod..

[26] Patel K.A., Brahmbhatt P.K. (2016). A comparative study of the RSM and ANN models for predicting surface roughness in roller burnishing. Procedia Technol..

[27] Lipinski D., Balasz B., Rypina L. (2018). Modelling of surface roughness and grinding forces using artificial neural networks with assessment of the ability to data generalization. Int. J. Adv. Manuf. Technol..

[28] Song H., Ren G., Dan J. (2019). Experimental study of the cutting force during laser-assisted machining of fused silica based on artificial neural network and response surface methodology. Silicon.

[29] Antil P. (2019). Modelling and multi-objective optimization during ECDM of silicon carbide reinforced epoxy composites. Silicon.

[30] Antil P., Singh S., Manna A. (2019). Experimental investigation during electrochemical discharge machining (ecdm) of hybrid polymer matrix composites. Iran. J. Sci. Technol. Trans. Mech. Eng..

[31] Finnie I. (1960). Erosion of surfaces by solid particles. Wear.

[32] Hutching I.M. (1992). Ductile-brittle transitions and wear maps for the erosion and abrasion of brittle materials. J. Appl. Phys..

[33] Kaundal R. (2014). Role of process variables on the solid particle erosion of polymer composites: A critical review. Silicon.

[34] Suresh A., Harsha A.P. (2006). Study of erosion efficiency of polymers and polymer composites. Polym. Test..

